# Multi-Host Transmission Dynamics of Schistosoma japonicum in Samar Province, the Philippines

**DOI:** 10.1371/journal.pmed.0050018

**Published:** 2008-01-22

**Authors:** Steven Riley, Hélène Carabin, Patrick Bélisle, Lawrence Joseph, Veronica Tallo, Ernesto Balolong, A. Lee Willingham, Tomas J Fernandez, Ryan O'Neal Gonzales, Remigio Olveda, Stephen T McGarvey

**Affiliations:** 1 Department of Community Medicine and School of Public Health, Li Ka Shing Faculty of Medicine, The University of Hong Kong, Hong Kong Special Administrative Region, China; 2 University of Oklahoma Health Sciences Center, Oklahoma City, Oklahoma, United States of America; 3 McGill University Health Center, Montreal, Quebec, Canada; 4 Research Institute for Tropical Medicine, Muntinlupa City, Republic of the Philippines; 5 World Health Organization/Food and Agriculture Organization Collaborating Center for Parasitic Zoonoses, University of Copenhagen, Frederiksberg, Denmark; 6 College of Veterinary Medicine, Visayas State University, Baybay, Leyte, Republic of the Philippines; 7 Brown University, Providence, Rhode Island, United States of America; Case Western Reserve University School of Medicine, United States of America

## Abstract

**Background:**

Among the 6.7 million people living in areas of the Philippines where infection with Schistosoma japonicum is considered endemic, even within small geographical areas levels of infection vary considerably. In general, the ecological drivers of this variability are not well described. Unlike other schistosomes, S. japonicum is known to infect several mammalian hosts. However, the relative contribution of different hosts to the transmission cycle is not well understood. Here, we characterize the transmission dynamics of S. japonicum using data from an extensive field study and a mathematical transmission model.

**Methods and Findings:**

In this study, stool samples were obtained from 5,623 humans and 5,899 potential nonhuman mammalian hosts in 50 villages in the Province of Samar, the Philippines. These data, with variable numbers of samples per individual, were adjusted for known specificities and sensitivities of the measurement techniques before being used to estimate the parameters of a mathematical transmission model, under the assumption that the dynamic transmission processes of infection and recovery were in a steady state in each village. The model was structured to allow variable rates of transmission from different mammals (humans, dogs, cats, pigs, domesticated water buffalo, and rats) to snails and from snails to mammals. First, we held transmission parameters constant for all villages and found that no combination of mammalian population size and prevalence of infectivity could explain the observed variability in prevalence of infection between villages. We then allowed either the underlying rate of transmission (a) from snails to mammals or (b) from mammals to snails to vary by village. Our data provided substantially more support for model structure (a) than for model structure (b). Fitted values for the village-level transmission intensity from snails to mammals appeared to be strongly spatially correlated, which is consistent with results from descriptive hierarchical analyses.

**Conclusions:**

Our results suggest that the process of acquiring mammalian S. japonicum infection is more important in explaining differences in prevalence of infection between villages than the process of snails becoming infected. Also, the contribution from water buffaloes to human S. japonicum infection in the Philippines is less important than has been recently observed for bovines in China. These findings have implications for the prioritization of mitigating interventions against S. japonicum transmission.

## Abstract 2

Obtaining schistosome infection data from thousands of humans and mammalian hosts in the Philippines, Steven Riley and colleagues find that mammalian acquisition, rather than transmission to snails, drives prevalence.

## Editors' Summary

**Background.:**

Parasitic worms called schistosomes infect about 200 million people worldwide. Different schistosome species are common in different parts of the world. Schistosoma japonicum, for example, is restricted to the Pacific region. In the Philippines, 6.7 million people live in areas where this parasite is endemic (constantly present). S. japonicum has a complex life cycle that starts with the parasite reproducing in freshwater snails. Free-swimming infectious parasites emerge from the snails and which burrow into the skin of people and other mammals (most schistosome species infect only people) when they swim in infected water. The parasites migrate to the veins draining the gut, where they mature into adult worms, mate, and lay eggs, some of which pass into the feces and back into the water where they hatch and infect fresh snails. Infection with S. japonicum does not kill many people but it causes serious health problems, including liver, lung, and gut damage.

**Why Was This Study Done?:**

Schistosome infections can be cured with inexpensive drugs, but people living in endemic countries usually become reinfected. The only way to avoid this problem is to eliminate the parasite in the environment, but to do so the relative contributions of snails, people, and other mammals to the transmission cycle need to be understood. Villages in the Philippines have very different levels of infection with S. japonicum. This variation suggests that the location of snail colonies and water courses and the behavior of the parasite's mammalian hosts (for example, their contact with water and their sanitary habits) affect the efficiency of S. japonicum transmission. Knowing which of these factors are the most important would help public-health officials instigate effective interventions to reduce infection rates. In this study, the researchers devise a mathematical model of S. japonicum transmission and use information on the number of people and other mammals infected in several villages in the Philippines to find out what drives inter-village variability in infection.

**What Did the Researchers Do and Find?:**

The researchers counted schistosome eggs in human and nonhuman mammalian feces collected in 50 villages in the Philippines (about 11,500 samples in total) and used these counts to estimate the parameters (values such as the rate of transmission from snails to people) for an S. japonicum transmission model. They then asked whether the proportion of the human population infected with S. japonicum predicted by the model matched actual data on infection levels in the villages. When they assumed that the transmission parameters were the same in all the villages, the variation in the number of mammalian hosts in each village could not explain the observed variation among villages in human infection levels. The researchers, therefore, tested two slightly more complex models in which the underlying rate of transmission from snails to mammals or from mammals to snails varied between villages to reflect different environmental conditions in each village. The first of these models fitted the available data best and also showed that cats, dogs, pigs, and water buffalo were less susceptible to infection with S. japonicum than people but that rats were more susceptible.

**What Do These Findings Mean?:**

These findings suggest that the snail-to-mammal side of the S. japonicum life cycle affects the inter-village variability in human schistosome infection more than the mammal-to-snail side. The findings also indicate that the contribution of water buffaloes to human S. japonicum infection in the Philippines is not particularly important. This contrasts with a recent study that identified water buffaloes as the major mammalian reservoir for S. japonicum in China. As with all mathematical models, the findings of this one depend on the assumptions made to build the model. Nevertheless, they suggest that interventions to reduce the size of the snail population and the exposure of mammals to parasite-containing water might reduce human infection levels more effectively than interventions that interrupt other parts of the parasite's life cycle. These results also suggest that further studies of the transmission of S. japonicum by water buffalo are needed before efforts are dedicated to treat or vaccinate water buffalo as a control measure against human S. japonicum infection.

**Additional Information.:**

Please access these Web sites via the online version of this summary at http://dx.doi.org/10.1371/journal.pmed.0050018.

- The MedlinePlus encyclopedia has a page on schistosomiasis (in English and Spanish)
- The US Centers for Disease Control and Prevention provides information for the public and professionals on schistosomiasis
- The World Health Organization provides information on schistosomiasis and research into its control

## Introduction

Modern, inexpensive pharmaceuticals are dramatically reducing the burden of disease caused by schistosomes and other waterborne macroparasites in resource-poor settings [1,2]. Many of the national programs delivering these anthelmintics are motivated and funded by large international aid initiatives. Given the high efficacy of the drugs and the increasingly recognized burden of morbidity and mortality imposed by these infections on the affected populations, such initiatives can be expected to represent good use of the large funds currently being made available by charities and governments for the control of neglected tropical diseases. However, even though some drugs such as praziquantel and albendazole are currently free or nearly free up to the point of importation into countries of use, their continued widespread distribution cannot be considered sustainable until such programs are independently prioritized by the health care systems of the populations that require them [3]. In addition, the mass delivery of such treatments is sometimes difficult. For example, during our project in 2004 in Samar province, the Philippines, only 49% of the population presented themselves for treatment with praziquantel, despite the treatment being provided free of charge. Given numerous competing local development objectives, it may be many years until countries such as the Philippines choose to prioritize the procurement and distribution of praziquantel in order to reduce the morbidity and mortality associated with Schistosoma japonicum infection.

An estimated 6.7 million people live in areas of the Philippines considered endemic for S. japonicum [4]. Mammals are infected by free-swimming larval forms of the parasite called cercariae. These larvae enter through the skin, and mature through different larval stages while circulating through the blood to the lungs before entering the hepatic portal system as mature males and females. There, they reproduce sexually, producing many eggs, which are discharged in the feces. Once in the environment, the eggs hatch and produce free-swimming miracidia, which infect amphibious snails from the genus *Oncomelania.* The miracidia reproduce asexually through sporocyst stages within these intermediate hosts, resulting in the production of many free-swimming cercariae. S. japonicum differs from other schistosomes by using other mammals in addition to humans as definitive hosts.

Human pathology ranges from mild to severe across several functional domains (e.g., liver disease and portal hypertension) and is associated mainly with an immune response to retained eggs, i.e., those not successfully discharged through the feces [5]. Single-dose chemotherapy with praziquantel is a highly effective treatment for S. japonicum infection. Currently, there is no viable human vaccine. Candidate water buffalo vaccines are the topic of ongoing investigation [6].

Village-level transmission dynamics of S. japonicum are greatly influenced by local ecology. In both China [7] and the Philippines [8,9], nearby villages often have very different infection profiles [10]. These infection profiles often return quickly to their baseline state after community-wide treatment with praziquantel [11]. The presence of an obligate free-swimming stage in the life cycle of S. japonicum suggests that the precise location of ecological features such as snail colonies, water courses, and rice irrigation canals can have a substantial impact on the efficiency of transmission. Furthermore, the behavior of local humans and reservoir species, especially in relation to their water-contact and sanitary habits, must also be important. Here, we use an information-theoretic approach [12] to compare multiple hypotheses concerning the transmission of S. japonicum within endemic villages in the Province of Samar in the Philippines. We use a simple deterministic mathematical model of S. japonicum transmission to formulate these various hypotheses and to compare their predictions with recently collected infection prevalence estimates.

## Materials and Methods

### Study Population

From one to three stool samples were obtained from nonhuman mammalian hosts (one sample, 731; two samples, 3,988; three samples, 1,181) and from humans (one sample, 1,582; two samples, 1,894; three samples, 2,148) in 50 villages in the Province of Samar, the Philippines, between August 2003 and November 2004. Humans were asked to provide stool samples on three consecutive days, but the actual number provided varied because of differences in compliance. We aimed to collect animal samples on at least two consecutive days, but numbers varied because small animals do not always defecate daily, and aggressive behavior in some animals prevented timely collection of samples. These villages were chosen from the 134 classified as endemic for schistosomiasis in Samar by the National Schistosomiasis Control Program of the Philippines. Because the initial study was designed to assess the impact of manmade irrigation on transmission, two groups of 25 villages were chosen. Although one group of villages was mostly rain-fed and the other was partially irrigated, no potentially important difference was found between the groups in the schistosomiasis infection profiles of humans [13] or of mammals [10].

Within each of the 50 villages, for human samples a maximum of 35 households were randomly selected from those households with at least five members and at least one farm worker. Criteria for inclusion mandated that the farm worker had to work full-time in a rice farm in the same category as that in which his village was classified (i.e., rain-fed or irrigated). Up to six people from each household were asked to provide three stool samples on consecutive days. For domestic animals, and for the first ten villages, all animals from each of the selected domesticated species (cats, dogs, pigs, and water buffaloes [i.e., the domesticated subspecies, the carabao]) were sampled. Because of logistical constraints, for the last 40 villages a census was taken and a random sample of 35 animals of each species was generated. For rats, 30 rat traps were set for 3 d. The location of the rat traps was changed each day to allow reasonable spatial coverage of each community within the time allowed. Stool samples were collected for one to three consecutive days.

Overall, at least one sample was obtained from 5,623 humans; 1,189 dogs; 1,275 cats; 1,899 pigs; 663 rats; and 873 water buffaloes. Further details about the study design are available for humans [8] and animals [10]. Note that in the analyses presented here, we omit data from one village in which no rat was trapped where 21 dogs, 14 cats, 27 pigs, 12 water buffaloes, and 168 humans were sampled.

### Parasitological Procedures and Correction for Measurement Error

We collected one, two, or three fecal samples from each human participant. Two 50 mg Kato-Katz slides were prepared from each human sample, refrigerated, and read by trained technicians. For animals, we collected either one, two, or three fecal samples. The Danish Bilharzia Laboratory method was used to estimate the concentration of eggs [14,15]. These two methodologies were used to obtain our observed eggs per gram (EPG) values for each fecal sample from each human or animal.

We used three infection classes for humans and two for animals. For humans, 0 EPG represented those uninfected, 1–100 EPG indicated light infection, and >100 suggested at least moderate infection. For animals, 0 EPG was taken as uninfected and >0 EPG as infected. However, the true infection status of an individual could not be inferred directly from the observed EPG values. For example, due to very low sensitivity and nonoptimal specificity of the Kato-Katz test, samples collected on consecutive days from the same human participant could fall into different infection classes. A similar issue arose for the Danish Bilharzia Laboratory method used for animal samples. Therefore, we used a simple Bayesian model to estimate the overall probability that each individual fell within a certain infection class given the known characteristics of the sampling methodology and the observed data from each participant. The statistical model used for this correction was identical to that described elsewhere [8,13] with the covariates for age, gender, household, village, and occupation removed. This adjustment for measurement error produced a set of posterior densities for the number of humans and animals of each type in each village, e.g., a posterior density for the number of at least moderately infected humans. We rounded the mode of these posterior densities to the nearest integer value and considered them to be our adjusted data.

### Transmission Model

**Figure 1:**
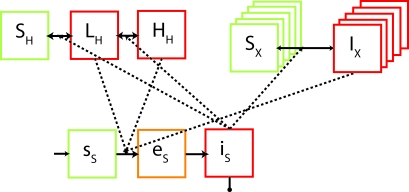
Structure of the Dynamic Model Subscripts denote species (*H*, human; *S*, snail; *X,* one of dog, cat, water buffalo, pig, or rat). Infection states are *E*, exposed and infected but not yet infectious; *H*, heavily infected; *I*, infected; *L*, lightly infected; and *S*, susceptible. Solid lines show the natural history of infection assumed for each species and dashed lines show the contribution by one species to the force-of-infection of another. The line below *I_S_*, terminated by a small circle, indicates the death of infected snails. We assume that the birth rate of susceptible snails is equal to the death rate of infected snails to ensure a constant snail population size.

We developed a mathematical model of S. japonicum transmission between humans, snails, and the five different animal host species (Figure 1). The force of infection at a given time in a transmission model is defined as the hazard of infection experienced by one susceptible individual as a result of all currently infectious individuals. We defined the number of humans in the *i*th village to be *N_H_*(*i*). Similarly, the number of animals from potential reservoir species was *N_X_*(*i*), where *X*∈(*C,D,P,R,W*} denotes the mammal species: cat (*C*), dog (*D*), pig (*P*), rat (*R*), and water buffalo (*W*). A door-to-door census was conducted in 2002–2003 by our research group for humans and all potential reservoir species [8,10] other than rats. We assumed that the size of rat populations in different villages was proportional to the number that were trapped. Due to the very dense water-course landscape in Samar, it proved difficult to obtain reliable estimates of the size of snail populations. Therefore, we model proportions of snails in each infection class, rather than actual numbers. All state variables described below are functions of time, and some transmission parameters are also functions of village index *j*. We omit the (*j,t*) notation where not necessary for convenience.

Humans in each village were classified as either susceptible *S_H_*, lightly infected *L_H_*, or heavily infected *H_H_*. The latter class could be more accurately described as at least moderately infected, i.e., moderate or heavy infection, but is referred to here as heavy infection. Other potential mammalian hosts were classified as either susceptible (*S_X_*) or infected (*I_X_*). The force of infection from mammals to snails in the *i*th village was defined as

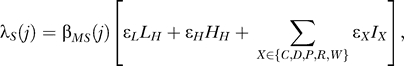
where *β_MS_*(*j*) was the underlying rate of transmission from mammals to snails in the *j*th village. The parameters *ɛ_L_* and *ɛ_H_* described the infectivity from humans with light infection to snails and from humans with heavy infection to snails respectively. The relative transmission from other mammalian hosts to snails was *ɛ_X_*, where *X*∈(*C,D,P,R,W*}. These relative infectivity parameters incorporated both behavioral and biological effects. For example, if mass of fecal output was a significant component of relative transmission during our study, larger animals such as water buffaloes would have had larger estimated values for infectivity parameters than smaller animals such as dogs. However, the village-to-village variation would not be affected by this scaling effect. Relative transmissions were assumed to be the same for all villages. Without loss of generality, we set *ɛ_L_* = 1. Effectively, the per capita infectivity of nonhuman mammals to snails and of heavily infected humans to snails were defined relative to that of lightly infected humans.

For a constant population size, the transmission dynamics of the snail population in each village were defined by the proportion of snails that were exposed but not yet infectious *e_S_* and the proportion of snails that were infectious *i_S_*. We defined the model by the time derivatives of its variables (using the notation 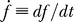 ),

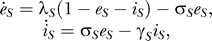
where *σ_S_* was the reciprocal of the average latent period in snails and *γ_S_* the reciprocal of the average infectious period in snails. Implicitly we assumed that infected snails die before recovery and were instantly replaced by susceptible snails. For values for *σ_S_*, we used the reciprocal of the midpoint of the range of latent periods reported for Oncomelania quadrasi infected with S. japonicum in Anderson and May [16] (5.9 years^−1^). For γ*_S_*, we used the average of the two life spans of O. quadrasi infected with S. japonicum reported in Anderson and May [16] (6.0 years^−1^). The proportion of snails that were susceptible, *s_S_*, was defined uniquely for all time by *s_S_* = 1 − *e_S_* − *i_S_*. However, for clarity of presentation, here we also define explicitly the proportion of snails that were susceptible,

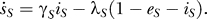



The infection dynamics of the human population were defined in terms of the numbers of lightly infected *L_H_* and heavily infected *H_H_* humans,

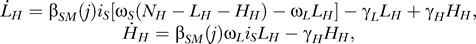
where *β_SM_*(*j*) was the underlying rate of transmission from snails to mammals in the *j*th village and there was no excess mortality of heavily infected humans (other parameters defined below). The number of humans that were susceptible, *S_H_*, was defined uniquely for all time by *S_H_* = *N_H_* − *L_H_* − *H_H_*. Here we also define the number of humans that were susceptible explicitly by its time derivative,

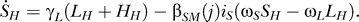



The parameters *ω_S_* and *ω_L_* described the relative transmission from snails to humans without infection and from snails to humans with light infection, respectively, and should be interpreted with some care (as should similar parameters for other species). These parameters are proportional to the hazard that infectious snails were able to cause humans to progress from one infection class to a higher class. However, a human's infection class did not correspond directly to a given number of established adult parasites. Hence, these parameters should not be interpreted as establishment rates. Without loss of generality, we set *ω_S_* = 1. A similar caveat applies to rates of recovery from infection. We defined *γ_L_* to be the reciprocal of the average time taken for a lightly infected human to become uninfected (as measured by Bayesian inferred infection class) in the absence of any additional infection. Similarly, *γ_H_* was the reciprocal of the average time taken for a heavily infected human to become lightly infected in the absence of any infectious challenge. These parameters do not reflect directly the average lifetime of a single adult parasite because one lightly infected human may have been infected with more than one mated pair. Similarly, one heavily infected human may have been infected with only a single adult parasite.

The dynamics of the mammalian reservoir species were defined by a single equation for *I_X_* for each species *X*∈(*C,D,P,R,W*},

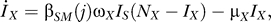
where *ω_X_* described the relative susceptibility of nonhuman mammals to infection by snails. The number of mammalian reservoir species that were susceptible, *S_X_*, was defined uniquely for all time by *S_X_* = *N_X_* − *I_X_*. Here, we also define the number of each mammalian reservoir species that were susceptible by its time derivative,

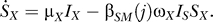



The parameter *μ_X_* was the reciprocal of the duration of infection of the reservoir species in the absence of additional infection. Because the adult parasites are long lived relative to the average lifetime of most reservoir mammals, this value was taken to be the reciprocal of the average lifetime of the mammal. Although this assumption is not as well suited to water buffaloes infected with S. japonicum as it is to the other hosts, it is likely still valid: our infectious class *I_W_* represented infectious water buffaloes with one or more mature mating pairs of adult parasites. Therefore, the expected time to cessation of infectiousness will be longer than observed average life spans of adult parasites (e.g., 3–5 y for S. mansoni [16]). We fitted exponential life times to the reported ages of cats (fitted mean value, 1.6 y), dogs (1.7 y), pigs (0.5 y), and water buffalo (8 y) to obtain values for the *μ_X_* parameters. We assumed that rats lived for 1 y on average.

To fit this dynamic model to the stationary cross-sectional data obtained from the study villages, we assumed that the dynamics were in a steady state (endemic equilibrium). This assumption was most likely met, as there was no indication that any of the villages had been subject to mass treatment or school-based treatment prior to the start of our study. It was straightforward to calculate numerically the steady state of this system of equations because they decoupled through the *i_S_* term. We used the asterisk notation (e.g., H_L_^*^) to denote equilibrium values of the state variables.

To calculate the likelihood of our adjusted data, given a solution of the transmission model, we assumed that the adjusted number of infectious individuals in each infection category (in each village for each species) was multinomially distributed with probabilities equal to the steady state prevalence, e.g., the number of humans followed a trinomial distribution with probabilities H_L_^*^/N_H_, H_H_^*^/N_H_ and 1 − (H_L_^*^ − H_H_^*^)/N_H_. Model solutions were obtained using the R statistical package [17]. The relative degree of support for different hypotheses was established using an approximate expression for the Akaike Information Criterion (AIC) [12]. We assumed that the estimate of prevalence for each host type was an independent sample. Therefore, 49 villages and seven host types gave *N* = 343. The AIC was equal to −2*L* + 2*K* + (2*K*(*K* + 1})/(*N* – *K* – 1) where *L* was the log likelihood and *K* the number of parameters (*N*/*K* < 40 [12]) (Table 1).

**Table 1:**
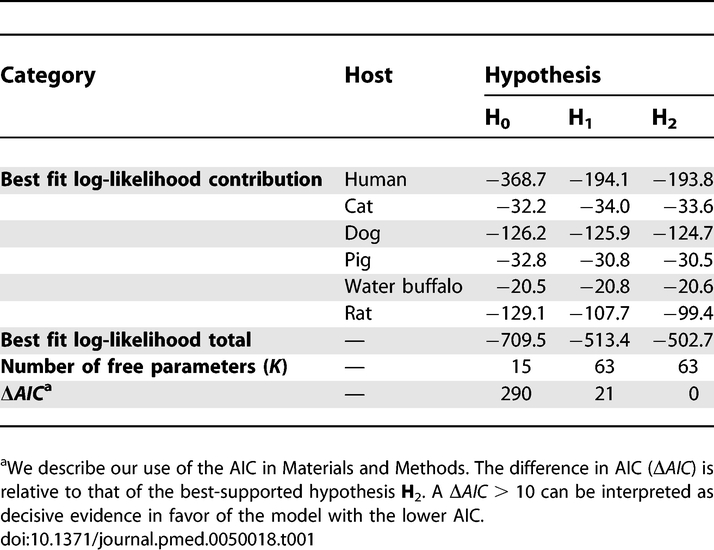
Comparison of Three Alternative Hypotheses to Explain between-Village Variation in Schistosoma japonicum Infection in Samar Province, the Philippines

## Results

**Figure 2:**
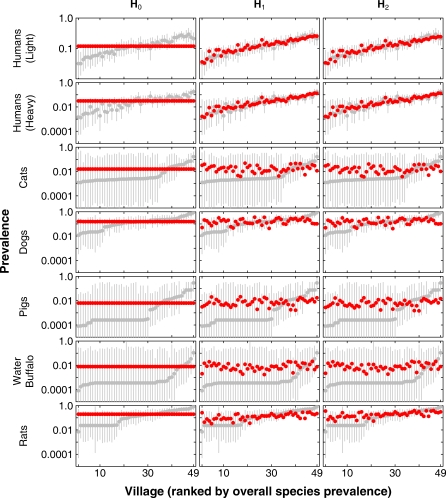
Comparison of Model (Red) and Observed (Grey) Prevalence Under Different Hypotheses for Two Classes of Human Infection and Five Potential Animal Reservoir Species Hypotheses were as follows. **H**_0_ (no site-specific variability, left column), **H**_1_ (site specific variability in process of infection from mammals to snails, centre column), and **H**_2_ (site specific variability in process of infection from snails to mammals, right column). Each of the 49 villages is indexed (x-axis) according to the prevalence of infection for each particular species, i.e., the ordering of villages is different for charts in different rows in this graph (other than top two rows). For humans (top two rows), the villages are indexed according to estimated overall prevalence. Grey points show the observed prevalence, adjusted jointly for the sensitivity and specificity of parasitological tests and the number of stool samples provided per participant. Binomial confidence intervals are presented for these adjusted data. The red points show the maximum likelihood steady-state values for the transmission model.

Initially, we assumed that there was no difference between the underlying transmission parameters in each village, i.e., for hypothesis **H**_0_ (Table 1), *β_MS_*(*j*) = *β_MS_*(*k*) and *β_SM_*(*j*) = *β_SM_*(*k*) for *j,k* ∈ 1…49. Therefore, the only source of variation in transmission was the different numbers of hosts of each species in each village. This model variant was not flexible enough to reproduce the observed variation in the data adjusted for measurement error (Figure 2). For each village, for a given class of infection prevalence, the values were very similar. This result suggests that it is not the distribution of potential mammalian reservoir and humans hosts in each village that drives the village-to-village variation in transmission. For example, there is no strong trend of increased infection in humans in those villages with a greater number of water buffaloes: this result is consistent with previous analyses based on a nonmechanistic model of these data [8].

For hypothesis **H**_1_ (Table 1), we relaxed our assumption that *β_MS_*(*j*) was equal for all villages, so *β_SM_*(*j*) = *β_SM_*(*k*) for *j,k* ∈ 1…49 but *β_MS_*(*j*) could take different values, i.e., we assumed that the rate of transmission from mammals to snails *β_MS_*(*j*) was site-specific [18,19]. This model choice corresponded to the presence of significant variation between villages in the defecation behavior and environmental dispersal of feces relative to the geographical location of snail colonies. Model variant **H**_0_ is a nested submodel of **H**_1_, so **H**_1_ was necessarily capable of achieving higher likelihoods. Furthermore, the difference in AIC between the two models shows strong support for **H**_1_ over **H**_0_. Hypothesis **H**_1_ produced reliable trends of infections in humans (and to a lesser extent rats) that were broadly consistent with the adjusted data.

For hypothesis **H**_2_, we relaxed the assumption from **H**_0_ that *β_SM_*(*j*) was equal for all villages, allowing it to take a different value for each village, while the rate of transmission from mammals to snails *β_MS_*(*j*) remained constant for all villages, *β_MS_*(*j*) = *β_MS_*(*k*) for *j,k* ∈ 1…49. This model choice corresponds to the presence of significant variation between villages in the following factors: the influence of local water courses in bringing cercariae into contact with humans, the water contact behavior of humans and other mammalian hosts, and the location of snail colonies along waterways relative to water contact sites of those hosts. Although the difference is not obvious in Figure 2, Table 1 shows that there is substantially more support for **H**_2_ over both **H**_1_ and **H**_0_ in the adjusted data. Hypothesis **H**_2_ was able to broadly reproduce both the correct trend and absolute values in prevalence of both lightly and heavily infected humans. The additional flexibility of **H**_2_ over **H**_1_ is that it was also able to better reproduce the observed trend of infection in rats in higher prevalence villages, which **H**_1_ was not able to do (Table 1). Note that in Figure 2, where confidence intervals are narrow for the adjusted data, small differences in model prevalence generated substantial differences in likelihood. Interestingly, although dogs did contribute substantially to calculated likelihoods, neither **H**_1_ nor **H**_2_ were able to give substantially better fits to these data compared with **H**_0_.

The interval estimates for the susceptibility of cats, dogs, pigs, and water buffaloes relative to lightly infected humans under **H**_2_ (*ω_S_* to *ω_W_* in Table 2) were all less than unity, i.e., humans were more susceptible to infection than any of these potential host species. This difference is as one might expect, given the high prevalence of infection in humans. However, given the much shorter life spans of animal reservoirs, this finding is still of interest. In contrast, rats were more susceptible than humans under **H**_2_. This high estimate of susceptibility of rats and the reduction in contribution to likelihood from rats when moving from **H**_1_ to **H**_2_ (Table 1) suggest that rats may play an important role in the transmission of S. japonicum in these villages. Note that the per capita estimate for rats should be treated with some caution because, for rats, the size of the population in the transmission model was assumed to be equal to the number trapped, which was almost certainly an underestimate. However, it seems likely that this approximation would affect estimates of infectivity more than estimates of susceptibility.

**Table 2:**
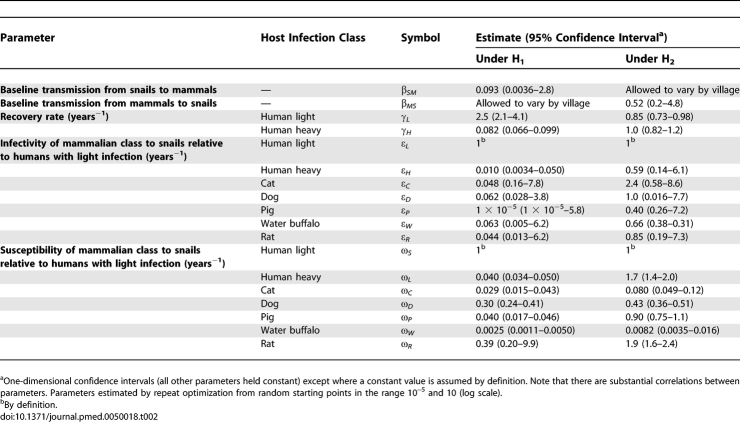
Estimated Parameter Values under Two Different Hypotheses in which One Transmission Parameter is Allowed to Vary at the Village Level

In the absence of comparably reliable data on snail infection prevalence, it is not surprising that the adjusted data contained little information on the infectivity of mammals relative to lightly infected humans (*ɛ_L_* to *ɛ_R_* in Table 2). The confidence intervals of these parameters were wide and generally included unity under **H**_2_.

**Figure 3:**
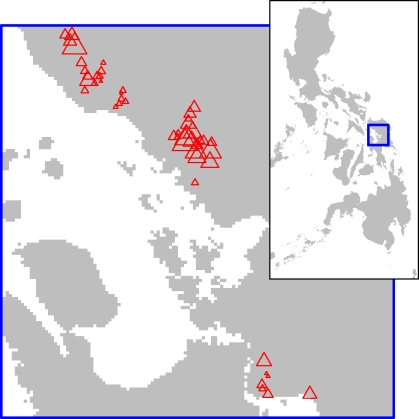
Spatial Distribution of the Underlying Rate of Transmission between Snails and Mammals *β_SM_*(*j*) under the Best Fit Hypothesis, **H**_2_ The linear size of the red triangles is proportional to the value of *β_SM_*(*j*). The outset chart shows the location of the study region in the Philippines. North is towards the top of the page in both charts.

The spatial distribution of estimated site-specific values for *β_SM_*(*j*) under **H**_2_ are shown in Figure 3, in which three distinct geographical areas can be seen. There is obvious spatial clustering of the larger values of *β_SM_*(*j*) in the middle-latitude area of villages and similar spatial clustering of the smaller values of *β_SM_*(*j*) in the southern area. This result is consistent with estimates of average human prevalence using descriptive spatial models [8].

## Discussion

We have used a simple transmission model to show that it is unlikely that variation in numbers of mammalian hosts is the main cause of variation in human S. japonicum infection in Samar, the Philippines. Inference using two slightly more complex model structures supports the hypothesis that site-specific variation in the underlying rate of transmission from snails to mammals is more important than site-specific variation in the underlying rate of transmission from mammals to snails. This result confirms and extends results from similar studies in China [18] in that a village-specific index of transmission seems likely to be driven more by one side of the parasite life cycle (the transmission from snails to mammals) than the other (the transmission from mammals to snails). Also, variation in the estimated rate of transmission from snails to mammals appears to be geographically clustered. Our results suggest that neither water buffaloes nor dogs contribute substantially to variation in infection rates, but that rats may be important.

Although rats may play an important role, without reliable estimates of the rat population sizes, it is difficult to quantify their importance in absolute terms. In the future we will attempt to estimate rat population numbers with the help of tropical agriculture experts. The inability of the transmission model to substantially improve its fit to the infection prevalence of dogs, even with the improved model flexibility of **H**_1_ and **H**_2_, contrasts with earlier findings (based on the same data, using a statistical model) [13] in which both dogs and cats were identified as potential contributors to the infection cycle. In general, transmission models differ from descriptive models by imposing additional correlations between variables that reflect assumptions about the mechanism of infection. Therefore, we suggest that our finding for dogs in this project may imply that infection in dogs does not contribute to the infection cycle in the same way that infection in rats does. For example, it is possible that some of the apparent infection in dogs may have resulted from ingestion of contaminated human feces, which would not directly contribute to infection. However, because we were not able to model explicitly age, sex, and occupation classes in this project, it is also possible that our finding for dogs is an artifact of village-to-village variation driven by differences in the human population that were not represented here.

Our results do not support water buffaloes as an important component of the S. japonicum transmission cycle that affects humans. However, the confidence intervals for our estimates of infection prevalence in all nonhuman, nonrat potential hosts are large. Therefore, we cannot rule out the possibility that there may be a small or moderate effect for water buffaloes that we failed to observe. Despite these uncertainties, our results are not consistent with a recent study of the impact of the treatment of water buffaloes in one of village in China [20], which suggests that these reservoir hosts may be responsible for as much as 75% of transmission compared with a single control village. Our findings here are consistent with the lack of a direct statistical association observed previously in these data [13]. One explanation is that substantial genetic variation in S. japonicum may exist between China and the Philippines. Such variation has been observed between schistosome miracidia found in the feces of different groups of hosts in China [21]. Therefore, the strain or strains contributing to infection in this study may be less well adapted for transmission in water buffaloes than the strains measured in the China study. Whatever the correct explanation for this apparent discrepancy, we suggest that the case in favor of the treatment or vaccination of water buffaloes in the Philippines as a control measure against human S. japonicum infection has yet to be made, and that more extensive studies of transmission in humans and water buffalo in China are warranted.

Our study was subject to a number of limitations. Because our principal interest was to investigate the interaction of different mammalian host species, we did not include human sex and age classes and occupation. This permitted a similar model structure for humans and other mammalian hosts and ensured that solutions to the transmission model could be obtained sufficiently quickly that parameter estimation was feasible. However, the inclusion of human age and sex classes and occupation would be desirable in future work and would allow the strength of site-specific effects (rather than population specific) to be estimated with more confidence. In contrast, our choice of two classes for infectious humans and one class for other mammals was driven by the accuracy with which the true infection status of individuals could be inferred. Previous statistical analysis suggests that two for humans and one for other mammals are the maximum number of infection classes that can be estimated with confidence [8,10]. If test sensitivity and specificity were improved, or many more samples obtained from each host, the framework we present here could be expanded to include more infection classes. Unfortunately, the methods used to measure snail density, which were applied alongside the field work described here, were not sufficiently reliable to be included in this quantitative study, as compared to the highly reliable census data available for other species. In addition, the data that were collected show very little variation in snail density from 147 sites where it was measured. This outcome is unfortunate, because we were forced to assume that there was no difference in the relative effective size of snail populations in different villages.

The feasibility of local elimination of S. japonicum in settings similar to those of our study has not yet been established. However, we suggest that the quantitative results presented here, and those from future studies (which will include individual-level data on treatment and follow-up), may help to make better use of available resources in reducing the burden of human infection prior to eventual elimination. We have shown that village-level variability in the process of mammalian infection, rather than snail infection, is better able to explain heterogeneity of S. japonicum prevalence which occurs between villages in the Samar Province of the Philippines. Therefore, for the purposes of reducing S. japonicum infection, more research should concentrate on estimating the cost-effectiveness of interventions aimed at reducing the size or competence of the intermediate snail population and reducing the exposure of mammals to water containing cercariae than on intervening upon other parts of the transmission cycle.

We hope to extend the scope of the results presented here in future work: there were a number of potentially interesting hypotheses that we were not able to investigate. For example, we did not allow more than one parameter to vary at the village level because of the substantial increase in computational complexity when moving from one-dimensional village-level optimization to two-dimensional. We will continue to investigate the use of more sophisticated mathematical approximations and optimization routines so that we can consider multiple village-specific parameters. Also, data from post-mass–treatment follow-up samples in the same villages will be available in the near future. With these data we hope to use the same inferential framework to quantify and explain any observed variability in the efficacy of mass treatment as an intervention.

More generally, explaining between-village variation in the prevalence of macroparasite infections is a common ecological problem [16]. Here we have assumed that a simple deterministic model was in a steady state and used large amounts of field data to test different hypotheses. We coped with hypotheses requiring model variants with large numbers of free parameters by using basic information theory to interpret results. This general approach could be used for other macroparasite diseases for which it is possible to measure some parts of the infection cycle accurately at many different locations.

## Abbreviations

AIC: Akaike Information Criterion
EPG: eggs per gram
